# Clinical relevance of splenic nodules or heterogeneous splenic parenchyma assessed by cytologic evaluation of fine‐needle samples in 125 dogs (2011‐2015)

**DOI:** 10.1111/jvim.15648

**Published:** 2019-11-06

**Authors:** Igor Yankin, Sarah Nemanic, Silvia Funes, Helio de Morais, Elena Gorman, Craig Ruaux

**Affiliations:** ^1^ Oregon State University Corvallis Oregon

**Keywords:** canine, FNA, spleen, splenic nodules

## Abstract

**Background:**

Splenic nodules and heterogeneous parenchyma are seen frequently in abdominal ultrasound examinations of dogs, but the clinical importance of these lesions remains unclear.

**Objectives:**

To determine whether specific ultrasonographic findings correlate with clinically relevant cytologic diagnoses and determine what sonographic features are correlated with these diagnoses. Another objective was to develop a scoring rubric to help clinicians make decisions on whether or not certain ultasonographic findings of the spleen warrant evaluation by fine‐needle cytology.

**Animals:**

One‐hundred twenty‐five adult client‐owned dogs with ultrasonographically identified splenic nodules, heterogeneous parenchyma, or both.

**Methods:**

Medical records were retrospectively searched for ultrasound‐guided splenic fine‐needle cytology reports. Ultrasonographic images were assessed for nodule number, size, echogenicity and distal enhancement, degree of splenic heterogeneity, and peritoneal fluid. Dogs were divided into 2 groups: those with clinically important or clinically irrelevant cytologic findings. Potentially useful and discriminatory ultrasonographic findings were identified by statistical analysis, and the most useful findings were used to generate the scoring rubric.

**Results:**

The clinically important group included 25 of 125 dogs (22 malignancies, 3 suppurative inflammation). Splenic nodules 1‐2 cm in diameter, peritoneal fluid, and >1 targetoid nodule were associated with clinically important cytologic findings. Receiver operator characteristic analysis showed that the scoring rubric was useful for identifying dogs in the clinically important group.

**Conclusions and Clinical Importance:**

Splenic fine‐needle cytologic findings identified a clinically relevant diagnosis in 20% of dogs, and larger nodule size, number of targetoid lesions, and presence of peritoneal fluid increase the likelihood of detection of clinically important disease.

**Abbreviations**
AUC: area under the curve
CI: confidence interval
ROC: receiver operator characteristic

## 1 INTRODUCTION

Sonographically detected splenic parenchymal changes can be found during abdominal ultrasound examination in asymptomatic patients or patients with unrelated disorders. Splenic nodules without associated splenomegaly are a relatively common finding in older dogs,^1^ are likely to be benign, and may require no further action in this age group.^1^ Nodular hyperplasia, hematoma, extramedullary hematopoiesis, congestion, and lymphoid hyperplasia are the most common non‐neoplastic lesions found in the spleen of dogs at necropsy or on biopsy,^2^, ^3^, ^4^ whereas the most common malignant neoplastic lesions of the spleen in dogs are hemangiosarcoma, histiocytic sarcoma, non‐angiomatous or nonlymphoid sarcomas, and lymphoma.^5^, ^6^ There is overlap in ultrasonographic appearance of benign and malignant splenic lesions, and they cannot be differentiated based on gray‐scale ultrasonography alone.^7^ Some studies found that neoplastic and non‐neoplastic splenic lesions had similar ultrasonographic appearance,^8^, ^9^, ^10^ whereas other studies found that specific appearances of nodules, such as targetoid nodules or multiple similar‐appearing nodules, more often were associated with malignancy and single lesions more often were benign.^11^, ^12^ Furthermore, diffuse parenchymal changes such as a “moth‐eaten” pattern resulting in moderately to severely heterogeneous splenic parenchyma can be seen in lymphoma and some other splenic disorders (e.g., systemic mast cell disease, leukemias, and splenitis) in dogs.^13^

Fine‐needle cytology is commonly performed in patients with splenic nodules, and fine‐needle sample collection is considered safe even in the presence of coagulopathies or thrombocytopenia.^11^, ^14^ In a study of human patients, splenic aspirates were diagnostically accurate in 85% (253/298) of the cases.^15^ In veterinary patients, splenic fine‐needle sample cytology has been reported to be as beneficial as needle core biopsy for diagnosing splenic neoplasia.^16^ In 1 study comparing the results of cytology and histopathology, splenic cytology correctly identified the underlying problem in 61% of cases, whereas histopathology was required to distinguish between neoplastic and reactive conditions in 23% of the cases.^11^ Similar results were reported in a study comparing cytological and histopathological diagnoses of splenic disorders in dogs, with a 59% agreement rate.^17^

The clinical relevance of small, nodular lesions in the spleen and the optimal clinical approach to patients with these lesions remains unclear. This is in contrast to patients with larger splenic masses (>2 cm diameter) or known underlying disease with a high rate of splenic involvement (e.g., mast cell tumor, lymphoma), in which it is generally accepted that fine‐needle aspirates of the splenic tissue are indicated.^18^

Our objective was to determine whether specific ultrasonographic findings correlate with a clinically relevant cytologic diagnosis and determine what sonographic features were correlated with these diagnoses. A further objective was to develop a scoring rubric to help clinicians make decisions on whether or not certain ultasonographic findings of the spleen warrant fine‐needle cytology.

## 2 MATERIALS AND METHODS

### 2.1 Case Selection

The medical records database of the Oregon State University Veterinary Teaching Hospital was retrospectively searched to identify dogs in which ultrasound‐guided splenic sample cytology was performed between July 2011 and June 2015. Criteria for inclusion were ultrasonographic examination of the spleen in which splenic nodules or heterogeneous splenic parenchyma were identified followed by splenic fine‐needle sample cytology on the same day. Nodules were defined as being <2 cm, whereas masses were defined as being >2 cm. In patients with splenic nodules identified on multiple visits, the ultrasound examination and cytology results from the first visit only were used in the study. The following criteria were used for exclusion: presence of a splenic mass (>2 cm), dogs known to have mast cell neoplasia or lymphoma before ultrasound examination, absence of a cytology report or ultrasonographic images, and dogs with homogenous splenic parenchyma without any nodules. Dogs with a diagnosis of lymphoma or mast cell neoplasia made during the first visit to the veterinary teaching hospital were not excluded from the retrospective analysis.

Information collected from medical records included signalment, date of imaging and sampling, presenting complaint, and cytologic diagnosis.

### 2.2 Procedures

Cytologic specimens were obtained by ultrasound‐guided fine‐needle sample collection performed by board‐certified veterinary radiologists. All ultrasonographic examinations and ultrasound‐guided sample collection was performed using an 8 MHz curvilinear transducer. Fine‐needle sample collection was performed using 22‐gauge, 1.5‐inch‐long needles using a nonaspiration technique.^19^, ^20^ A needle was guided into the spleen and then gently moved up and down along the needle tract in an attempt to harvest cells; no negative pressure was applied to the syringe.^20^ Splenic nodules that had characteristic ultrasonographic appearance of myelolipomas were not routinely sampled.^21^, ^22^ All ultrasonographic studies were performed by board‐certified veterinary radiologists using the same ultrasound machine (iU22 Ultrasound System; Phillips Medical Systems, Bothell, Waltham). Slides for cytologic evaluation were prepared by making smears with a feathered edge and then staining with a modified Wright‐Giemsa stain (Richard‐Allan Scientific; a Hema‐Tek 2000 Slide Stainer, Model 4488, Bayer Corporation). Smears were prepared immediately after sample collection and stained within 1 hour post‐submission.

Static images of ultrasound examinations and radiology reports were reviewed by 2 authors (I.Y., S.N.), an intern and a board‐certified veterinary radiologist to determine the ultrasonographic appearance of the splenic parenchyma and nodules. Observers were blinded to the clinical presentation, history, and cytology results. Information collected from ultrasonographic images and radiology reports included the number and size of the largest nodules, presence of distal acoustic enhancement of the nodules, echogenicity of nodules, margination of the nodules (ill‐ or well‐defined), heterogeneity of the splenic parenchyma, and presence of peritoneal fluid.

Targetoid lesions were defined as nodules with a hypoechoic rim and a hyperechoic or isoechoic center.^12^ In cases with multiple nodules, the size determination was based on the largest nodule.

Board‐certified clinical pathologists reviewed all cytologic slides for diagnostic interpretation. The retrospective review of the cytologic slides was performed by an independent board‐certified clinical pathologist (E.G.) in a blinded fashion without knowing the size and characteristics of the nodules and without knowledge of the initial diagnostic interpretation. Lesions were interpreted based on the predominating cellular constituents present. Nodules considered to represent a benign or normal process included extramedullary hematopoiesis (presence of mixed hematopoietic precursors and occasional hemosiderophages), lymphoid and plasma cell hyperplasia or reactivity, or no abnormalities based on the presence of normal blood constituents with no cellular proliferation. These findings were classified as clinically irrelevant because they did not affect case management. Cytologic diagnoses of malignant neoplasia were based on the presence of abnormal cellular infiltrates (atypical spindle or epithelial cells) or proliferation of monomorphic populations of atypical round cells including lymphocytes, histiocytes, or mast cells. Interpretation of inflammation was based on increased numbers of leukocytes in excess of expected numbers based on known peripheral blood results. Neoplastic and inflammatory lesions were classified as clinically relevant if they changed case management. Based on the clinical importance of the cytologic diagnoses, dogs were divided into 2 groups (clinically important versus clinically irrelevant), and ultrasound findings were compared between groups. Group 1 was comprised of dogs with clinically important cytological diagnoses, whereas group 2 included dogs with clinically irrelevant cytologic diagnoses.

A “clinical importance score” rubric (Table 2) was created based on the ultrasonographic findings and their statistical relevance (Table 1). For each of these findings, a specific score was given with a maximum possible total score of 13 points.

**Table 1:** Ultrasonographic findings in dogs with splenic nodules, heterogeneous splenic parenchyma, or both

Variable	Group 1 (number of dogs)	Group 2 (number of dogs)	*P* value	Test
Number of nodules				
No nodules	1/25	15/100	**.07**	Chi‐squared test for trend
1	3/25	19/100		
>1	21/25	66/100		
Size of nodules, cm				
1‐2	21/24	47/85	**.01**	Fisher's exact test
<1	4/24	38/85		
Presence of distal enhancement of nodules	3/25	3/100	**.09**	Fisher's exact test
Nodule echogenicity				
Hypoechoic	15/25	59/100	1	Fisher's exact test
Hyperechoic	2/25	15/100	.52	
Mixed echogenicity	4/25	7/100	.23	
Targetoid	3/25	4/100	**.14**	
Nodule margins				
Well‐defined	9/24	23/100	.32	Chi‐squared test for trend
Ill‐defined	15/24	62/100		
Splenic parenchymal heterogeneity				
Homogenous	7/25	26/100	.28	Chi‐squared test for trend
Mildly heterogeneous	4/25	25/100		
Moderately heterogeneous	7/25	40/100		
Severely heterogeneous	7/25	9/100		
Presence of targetoid nodules				
1	no	4/100	**.01**	Fisher's Exact Test
>1	3/25	no		
Presence of peritoneal fluid	12/25	19/100	**.002**	Fisher's Exact Test

Note: Bolded values are indicative of a *P* value of less than 0.05.

### 2.3 Statistical analysis

**Figure 1:**
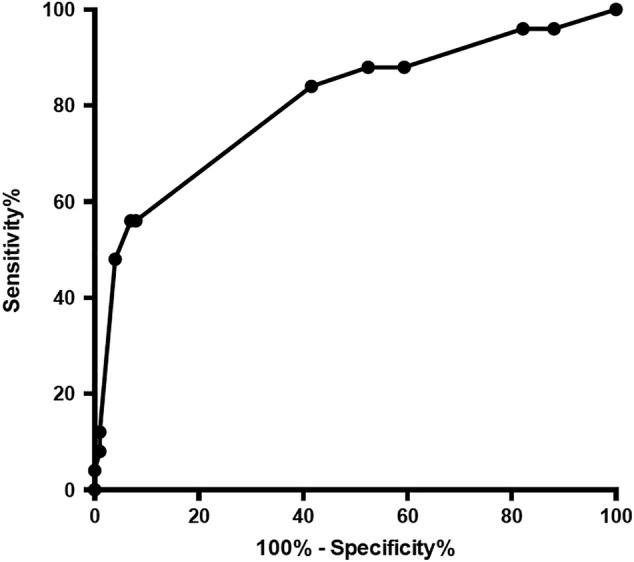
The receiver operator characteristic (ROC) analysis. The ROC analysis showed an area under the curve (AUC) of 0.804, with a 95% confidence interval (CI) for AUC of 0.697 to 0.911, *P* < .0001. This indicates that this scoring system is significantly better than random chance (AUC of 0.5) at detecting clinically important disease in the spleen

For analysis, dogs were classified into group 1 or group 2 based on the cytologic diagnosis and its impact on the management of the case. Potential marker findings for clinically relevant changes initially were analyzed using contingency tables. Binary variables (e.g., nodules present, peritoneal fluid present) were analyzed using Fisher's Exact Test, whereas variables that were stratified (e.g., parenchymal heterogeneity, total nodule number) were analyzed using the chi‐squared test for trend. For the initial univariate analyses, statistical significance was set at *P* < .05. After univariate analysis, a multiple factor “clinical importance score” rubric was created. Briefly, variables from the univariate analysis that showed a calculated *P* value < .1 were identified, and numeric scores assigned for each finding. Scores were weighted by relative risk identified in the contingency analysis, with findings that had a high relative risk for the presence of clinically relevant disease given a higher score. For variables that were stratifiable, increasingly more severe findings were given higher weightings in the scoring system. The scoring system was applied to the ultrasonographic data, and the diagnostic performance of the scoring system assessed using receiver operator characteristic (ROC) analysis (Figure 1). The scoring system then was simplified by stepwise exclusion of lower significance findings from the univariate analysis and comparison of the areas under the curves (AUC) for the ROC analyses using the 95% confidence intervals (CI) of the resulting curves. If removal of a variable significantly decreased the diagnostic utility of the scoring system, the variable was retained.

All statistical analyses were performed using GraphPad Prism 6.0 for the Macintosh operating system (GraphPad Prism 6.0; GraphPad Software, La Jolla, California).

## 3 RESULTS

The medical records search identified 245 dogs meeting the initial inclusion criteria. One‐hundred sixteen dogs were excluded from the study: 64 dogs were diagnosed with mast cell tumor or lymphoma before splenic fine‐needle sample cytology, 45 dogs had a mass ≥ 2 cm in diameter, 6 dogs did not have cytology reports or ultrasound images available, 1 dog had homogenous splenic parenchyma without any nodules, and 1 dog had a nondiagnostic sample. These excluded cases resulted in a sample size of 125 dogs that met the final criteria for assessment.

Group 1 included 25 of 125 (20%) dogs with clinically important cytologic diagnoses including lymphoma (n = 10/25), sarcoma (spindle cell) tumor (n = 4/25), malignant histiocytosis (n = 3/25), suppurative inflammation (n = 3/25), epithelial neoplasm (carcinoma; n = 3/25), melanoma (n = 1/25), and hemangiosarcoma (n = 1/25). Group 2 included 100 of 125 dogs (80%) with cytologic diagnoses not impacting the diagnostic or therapeutic plan, including extramedullary hematopoiesis (n = 79/100), lymphoid reactivity or hyperplasia (n = 12/100), and normal spleen (n = 9/100).

The mean age of dogs in group 1 was 9.0 years (range, 5 months to 13 years), whereas the mean age of dogs in group 2 was 9.4 years (range, 9 months to 18 years); no significant difference was found in age between the 2 groups (*P* = .62). The most common breeds of dogs in group 1 were mixed breed dogs (6/25), Golden Retriever (4/25), and Labrador Retriever (4/25). The most common breeds of dogs in group 2 were mixed breed dogs (25/100), Golden Retriever (9/100), Labrador Retriever (7/100), Dachshund (5/100), Flat‐Coated Retriever (3/100), Heeler (3/100), Boxer (3/100), and German Shepherd Dog (3/100). Of the 25 dogs in group 1, 17 were neutered males, 7 were spayed females, and 1 was a sexually intact female. Of the 100 dogs in group 2, 38 were neutered males, 2 were sexually intact males, 55 were spayed females, and 5 were sexually intact females. No statistical difference in breed or sex was found between the groups.

The presence of splenic nodules with diameter 1‐2 cm was significantly more common in dogs with clinically important splenic lesions (21/25 dogs versus 47/85 dogs in group 2; *P* = .01). A higher prevalence of peritoneal fluid was found in dogs with clinically important splenic lesions (12/25) when compared to dogs with clinically irrelevant lesions (19/100, *P* = .002). Three of 25 dogs in group 1 had multiple targetoid nodules, and no dogs in group 2 had multiple targetoid nodules, but 4 of 100 dogs in group 2 had singular targetoid nodules (*P* = .01). The presence of multiple nodules was not different between groups (*P* = .07). Other findings are summarized in Table 1.

The scoring rubric differentiated irrelevant from clinically important splenic lesions, as shown in Table 2. A cutoff value of >5 resulted in a sensitivity of 56% and a specificity of 92% in this patient population (Table 2). A cutoff value >4 resulted in an increased sensitivity of 84% and decreased specificity of 58%. The ROC analysis showed an AUC of 0.804, with a 95% CI of 0.697 to 0.911 (*P* < .0001; Figure 1).

**Table 2:** Scoring system for the likelihood of achieving a cytologic diagnosis that will affect case management based on ultrasonographic findings

Variables	Score	Sensitivity (>5^a^)	Specificity (>5)	Sensitivity (>4)	Specificity (>4)
Number of nodules	0 = 0, 1 = 1, >1 = 2				
Nodule size, cm	0 = 0, <1 = 1, 1‐2 = 2				
Presence of distal enhancement	Any enhancement = 2	56%	92%	84%	58%
Presence of targetoid nodules	Any targetoid nodules = 1				
Number of targetoid nodules	>1 targetoid = 2				
Presence of peritoneal fluid	Fluid present = 3				

a A cutoff value of total score.

## 4 DISCUSSION

Our results showed that malignancies, particularly lymphoma, were the most common cytologic findings in dogs with clinically relevant diagnoses. Significant differences in the size of nodules and the presence of peritoneal fluid were found between the groups. The presence of multiple nodules had a *P* value < .1 between the groups and was incorporated into the overall clinical importance scoring system. No significant differences in age, breed, and sex of dogs as well as splenic parenchymal heterogeneity, margination of nodules, distal enhancement of nodules, and nodule echogenicity were found between the 2 groups.

In our study, 100 of 125 dogs had benign splenic pathology or normal splenic tissue, as interpreted by cytologic evaluation. The ratio of non‐neoplastic to neoplastic splenic disease in dogs varies among previous studies. Studies that included all cases of splenomegaly or masses showed >50% prevalence of non‐neoplastic diseases.^1^, ^2^, ^11^

We found that the presence of splenic nodules 1‐2 cm in diameter was predictive of a clinically important cytologic diagnosis. A change in diameter from 1.0 to 1.25 cm is associated with a doubling in volume for a spherical mass,^21^ and based on these findings, we recommend performing fine‐needle cytology of the spleen in dogs with splenic nodules 1‐2 cm in diameter.

In our study, the presence of peritoneal fluid was more common in dogs with clinically important splenic lesions. Any amount of peritoneal fluid was considered a positive finding, including amounts that were not amenable to abdominocentesis. Thus, the type of fluid was not assessed in all cases and not evaluated in our study. Overall, 12 of 25 dogs with a cytologically important diagnosis had sonographically detectable peritoneal fluid compared to 19 of 100 dogs in group 2. Although the presence of peritoneal fluid in dogs with splenic masses often is associated with neoplasia,^6^ ours is the first report highlighting the importance of peritoneal fluid in dogs with splenic nodules. Based on these findings, we recommend performing fine‐needle sample cytology of the splenic parenchyma of dogs in which peritoneal fluid is seen in conjunction with either splenic nodules or splenic parenchymal heterogeneity.

For the dogs described here, the presence of multiple splenic nodules was a risk factor for having a clinically important cytologic diagnosis, using the scoring rubric. These results were similar to those of a previous study, where the finding of multiple discrete lesions with similar ultrasonographic appearance was significantly associated with malignancy, and single lesions more often were benign.^11^

Three of 25 dogs in group 1 had multiple targetoid nodules, and no dogs in this group had singular targetoid nodules. Only 4 of 100 dogs with clinically irrelevant cytologic diagnoses had singular targetoid nodules, and no dogs in this group had multiple targetoid lesions. Multiple targetoid nodules could be suggestive of clinically relevant splenic pathology. In a previous study evaluating singular and multiple targetoid nodules in the spleen and liver, 17 of 23 singular lesions and 13 of 16 multiple lesions were associated with a malignant neoplasm.^12^ In our study, the presence of >1 targetoid lesion was associated with increased relative risk for a clinically important diagnosis on splenic sample cytology, but the number of animals having >1 targetoid lesion (n = 3 dogs, all in group 1) was low, and these results should be interpreted with caution.

The scoring system described here is based on the combination of ultrasound findings that showed potential utility for the presence of a clinically important disease. A score > 5 is suggestive of clinically important ultrasonographic findings with an excellent specificity of 92%, but poor sensitivity of 56%. Decreasing the cutoff score to >4 improves the sensitivity of the scoring system to 84% but decreases its specificity to 58%. The AUC of the ROC analysis of 0.804 (95% CI, 0.697‐0.911) indicates that this scoring system is significantly better than random chance (AUC of 0.5) at detecting clinically important disease in the spleen (Figure 1). Our results suggest that splenic fine‐needle sample cytology should be considered in dogs with a score >4‐5.

As with most retrospective studies, our study had some limitations. The first limitation was the absence of histopathologic confirmation of the cytologic diagnosis. Although cytologic diagnoses often reflect histologic results, if incorrect or inadequate sampling occurs or cytology is unable to distinguish between reactive and neoplastic conditions, accurate diagnosis with fine‐needle sample cytology may not be possible.^11^, ^15^ To minimize the possibility of error, a blinded retrospective review of the cytologic slides was performed by an independent clinical pathologist. Excellent agreement was found between the second slide review and the initial evaluation that did not change the results of the statistical analysis. Our study, however, was not intended to compare or evaluate diagnostic accuracy of cytology as compared to histopathology but to determine whether specific ultrasonographic findings correlated with a clinically important cytologic diagnosis.

A second limitation also is related to the retrospective nature of the study. It was not definitively documented that the splenic fine‐needle cytology sample was obtained from the nodules or surrounding parenchyma. It is our general procedure to sample nodules as well as some non‐nodular parenchyma, but we cannot be sure that this was always the case.

A third limitation relates to the retrospective evaluation of the static ultrasonographic images of the spleen and radiology reports because only saved images could be evaluated and images may not have been acquired of all nodules. All ultrasound examinations were performed by 1 of 2 board‐certified radiologists, and multiple images of the spleen were available for all animals. The combination of this consistency combined with a radiology report written within 24 hours of the ultrasound examination and the presence of static images documenting the appearance of the spleen decreases the possibility that the ultrasonographic data incorrectly represented the disease of the patient.

In conclusion, splenic fine‐needle sample cytology is a minimally invasive diagnostic modality that identified a clinically important diagnosis in approximately 20% of adult dogs with splenic nodules or heterogeneous splenic parenchyma. Specific ultrasonographic findings, such as the size and number of splenic nodules as well as the presence of peritoneal fluid, may increase the index of suspicion for the presence of malignant neoplasm or suppurative inflammation. Use of the proposed scoring method and a cutoff score of either 4 or 5 may help identify which animals in this population of patients may benefit the most from splenic fine‐needle sample cytology after ultrasound examination.

## CONFLICT OF INTEREST DECLARATION

Helio de Morais serves as Associate Editor for the *Journal of Veterinary Internal Medicine*. He was not involved in review of this manuscript.

## OFF‐LABEL ANTIMICROBIAL DECLARATION

Authors declare no off‐label use of antimicrobials.

## INSTITUTIONAL ANIMAL CARE AND USE COMMITTEE (IACUC) OR OTHER APPROVAL DECLARATION

Authors declare no IACUC or other approval was needed.

## HUMAN ETHICS APPROVAL DECLARATION

Authors declare human ethics approval was not needed for this study.
